# A Calcarea Case

**Published:** 1899-04

**Authors:** J. E. Huffman

**Affiliations:** Healdsburg, Cal.


### A CALCAREA CASE.

J. E. Huffman, M. D., Healdsburg, Cal.

September 9, 1895—John B., colored, San Francisco, æt. twenty months. Large head; ant. fontanelle, size half a dollar; irritable; cries when spoken to; enlargement of end of radius and ulna; large abdomen; legs crooked; thirsty, cries in night for water; eight teeth; can stand only when has hold of chair, and then not straight; prepuce adherent. ℞ Calc-carb., 50 M.

September 16th.—Broke up adhesions of prepuce, became much better and sleeps all night.

October 4th.—Has a cold, irritable, and eruption behind ears. ℞ Calc-carb., 50 M.

December 11th.—Stands alone; fontanelle closing rapidly. ℞ Calc-carb., 50 M.

Did not see him again. Parents moved to Portland in February, 1896, but I learned afterward that he was walking well at time of removal. I treated him while I was a student. I gave S. L. between doses of medicine.

